# Employer impact on COVID-19 vaccine uptake among nursing and social care employees in Austria

**DOI:** 10.3389/fpubh.2022.1023914

**Published:** 2022-11-10

**Authors:** Ann-Kathrin Ruf, Sabine Völkl-Kernstock, Magdalena Eitenberger, Marcus Gabriel, Elisabeth Klager, Maria Kletecka-Pulker, Sophie Klomfar, Anna Teufel, Thomas Wochele-Thoma

**Affiliations:** ^1^Ludwig Boltzmann Institute Digital Health and Patient Safety, Vienna, Austria; ^2^Medical University of Vienna, Department of Child and Adolescent Psychiatry, Vienna, Austria; ^3^Institute for Ethics and Law in Medicine, University of Vienna, Vienna, Austria

**Keywords:** COVID-19, COVID-19 vaccine, vaccine hesitancy, vaccine incentives, employer impact

## Abstract

**Introduction:**

Since becoming available, vaccines against COVID-19 have been a focus of public debate. This is particularly relevant among healthcare and social workers, who interact with vulnerable patients and clients on a daily basis. With employers implementing educational programs and offering incentives to raise vaccine willingness among their staff, it is crucial to understand drivers of vaccine acceptance and hesitancy as well as the impact employers can play on vaccine decision-making.

**Methods:**

We conducted a cross-sectional study *via* computer-assisted telephone and web interviews. We recruited from a pool of employees from nursing and social care institutions in Vienna and Lower Austria operated by one healthcare NGO. Variables included in the analysis were socio-demographic attributes, reasons for or against the vaccine, sources of information, opinions of mandatory vaccination, and whether respondents had previously been infected with COVID-19 or knew someone who had.

**Results:**

86.2% of respondents had received at least one dose of the COVID-19 vaccine. 13.8% were unvaccinated. Vaccinated respondents' main reason for getting the vaccine was to protect themselves (79.6%) as well as others (74.1%), while non-vaccinated respondents cited a fear of short or long-term side effects (58.8 and 42.4%, respectively) as their primary reason for not getting vaccinated. 72.8% of the unvaccinated said no incentive would make them change their mind, while 17.4% specified abstract concepts or systemic change as effective incentives. Monetary incentives were not seen as a motivator. Unvaccinated respondents were significantly more worried about the future than vaccinated respondents (78.8 vs. 26.3%, *p* < 0.001). They were also significantly more likely to view their employers' vaccine recommendations as “manipulative” (50.6 vs. 12.4%, *p* < 0.001), while vaccinated respondents were significantly more likely to view them as “supportive” (68.0 vs. 25.9%, *p* < 0.001).

**Conclusion:**

While employers have the means to mediate public health decision-making by providing information, deciding to become vaccinated is a more complex process including public debate, world views, political influences, and the uptake of information. Employers can act as mediators for public health decision-making, moving policy measures beyond an individualized view of health choices and health literacy toward more structural, systemic, and community-based efforts.

## Introduction

Ever since vaccinations for COVID-19 became available in December 2020 they have been a focus of public debate. Following initial excitement about finally having an effective tool against the pandemic and a first focus on protecting healthcare workers and vulnerable populations, it quickly became apparent that many did not view the vaccine as the panacea it set out to be (1). With discourse around the safety and efficacy of vaccines becoming increasingly heated, compulsory vaccination mandates and other measures to increase vaccination rates were discussed as possible public health measures (2, 3). Meanwhile, healthcare and social care providers sought different methods of encouraging employees to get vaccinated, arbitrating between notions of freedom of personal choice on the one hand and, on the other, employees' personal protection as well as that of their patients and clients (4, 5).

Against the backdrop of this increasing tension, our study focuses on vaccination attitudes amongst employees of one large nursing and social care NGO based in Vienna and Lower Austria. As of October 2021, over 80% of the 6,000 nursing and social care employees had been voluntarily vaccinated against the virus and, with COVID-19 cases on the rise, the institution was looking for ways to increase this figure. At the time, Austria's vaccination rate lagged behind that of many other European countries: 75% of people in Austria had received at least one dose compared to 93% in Portugal, 84% in Spain, 83% in Italy, 82% in Denmark, and 80% in Norway and Ireland (6). Furthermore, the press reported that even a notable number of healthcare workers were skeptical of vaccinations and vaccination mandates (7). Austria was about to enter its third national lockdown and gained international press attention by ending the lockdown early for people who had been vaccinated or had recently recovered from COVID-19, de facto indirectly penalizing the non-vaccinated (8). Unvaccinated healthcare workers were especially harshly criticized, making the topic of getting vaccinated for the sake of patient and client safety the focus of debate (9).

Our survey, conducted in December 2021, explores the reasons and justifications given by nursing and social care employees at one Austrian healthcare NGO for receiving or refusing a COVID-19 vaccination. In computer-assisted web and telephone interviews, we asked respondents which sources they used to gather information, and what might incentivize non-vaccinated employees to change their mind and be vaccinated. The NGO in question had also been particularly active with respect to educating and informing unvaccinated employees about the vaccinations, the risks to their employees' own health, and to the health of their clients. In recent studies, the role of employers in vaccine decision-making and shaping opinions about vaccination policy measures has been repeatedly emphasized (10–12). Lazarus et al. (13) examined international differences, surveying respondents in 23 countries and asking how they would respond to an employer's hypothetical recommendation that they get vaccinated. The authors found that employers can, at least hypothetically, play an important role in mediating vaccination decision-making, and differences among countries indicate potential cultural and structural effects. This study provides a more detailed test of this hypothetical scenario in the context of Austria.

We chose to conduct our research at this specific healthcare NGO because it had been using additional measures to incentivize employees to get vaccinated as early as January 2021, the date at which the general vaccine rollout started in Austria. By the time this study was conducted, employees had consequently received a wealth of information about the benefits and possible risks of the vaccine through their workplace and had been given further opportunities to seek more information. Employees were also regularly tested for COVID-19 to avoid the spread of infection amongst high-risk clients such as older adults and persons living with physical disabilities for whom a large number of employees had care and nursing duties (14). To encourage employees to become vaccinated, managing directors decided to introduce individual, mandatory consultations with a physician about the vaccine. Non-German speakers were able to receive their consultation in their native language using tele-interpreters (15). Additionally, managing directors, employee representatives and company health officers decided to jointly send an informational letter to non-vaccinated employees. The letter addressed potential fears and concerns about the vaccine, emphasized the risk that a COVID-19 infection poses to the employee's own health and that of their patients and clients, and mandated these employees to visit the information line at one of the local vaccination centers where doctors were specifically tasked and trained to provide further information. Despite these elaborate efforts, only 5% of non-vaccinated employees opted for a vaccination following such a consultation.

Overall, this points to deeper-seated issues structuring people's attitudes and justifications, making this a particularly pertinent issue among people whose job involves patient care. A recent study of the attitudes of midwives in Austria to measles vaccinations has shown that information and education alone do not change vaccination attitudes (16). In our study, we point to the role played by the workplace: both the potential role of broad reaching but subnational (community) efforts, and what employers can and cannot do to effectively incentivize vaccinations as a means of achieving overarching public health goals.

In our discussion, we ask how communication and policy strategies can reach unvaccinated healthcare NGO workers—and non-vaccinated people in general—in a country where a large range of relevant policy measures has already been exhausted, including the world's first vaccine mandate (17)^1^.

## Materials and methods

The study's main aim was to evaluate the reasons given by employees of several nursing service providers in Vienna and Lower Austria for receiving or refusing a COVID-19 vaccination. An additional aim was to identify their sources of information about the vaccine.

### Design, subjects and procedure

This was a cross-sectional study conducted *via* computer-assisted telephone and web interviews. We recruited from a pool of employees at nursing and social care institutions in Vienna and Lower Austria, all operated by a single healthcare NGO.

After approving our planned survey, the employer provided us with a list of 6,033 employee work telephone numbers. Of these, 360 numbers were selected at random for calling. Six researchers conducted telephone interviews during the period December 20 to 23, 2021. During this time, we called each number at least once. *n* = 36 persons agreed to be interviewed. *n* = 238 persons answered the phone but were not willing to participate or asked to be called back and then did not answer their phone again. Where we were unable to reach a respondent (i.e., when their phone was switched off, or we only reached their mailbox), we called them again later. In 86 cases, no one answered the phone despite multiple attempts to call.

After 4 days of cold calling, our response rate was 10%. With Christmas and New Year's Eve approaching, we were not optimistic that we would reach significantly more people in the following days. We therefore decided to host the survey online and issue an email to all employees asking them to participate. Data was collected *via* the online survey from December 27, 2021, to January 10, 2022.

To prevent those who had already participated in the telephone survey from also participating online, we began the survey with the question: “In the last couple of days, have you taken part in a telephone interview about the COVID-19 vaccine?” Respondents who answered “yes” to this question were screened out. After screen out, *n* = 589 respondents completed the online survey. None needed to be excluded for quality reasons.

### Measurements

We collected respondents' demographic data with respect to gender, year of birth, level of education, and country of birth. The levels of education included in the demographic data are specific to the Austrian school system and consist of “compulsory school or lower,” indicating 9 years of obligatory education usually completed at age 15, “apprenticeship,” which is a practical professional training, “leaving certification,” the equivalent of a high school diploma, and “university / university of applied sciences,” which corresponds to any kind of higher education after high school. Today, most nursing practitioners and social care workers have university-level degrees and therefore fall in the category of “university or applied sciences” (or similar diploma) category^2^.

While designing the survey, we also debated including more detailed questions regarding characteristics of employees, such as exact profession (e.g., nurse/social care worker) or years of employment at the NGO. However, we opted against these data points in order to protect participant anonymity, as the political pressure for healthcare-related workers to get vaccinated was at an all-time high when we conducted the study.

The survey's main section began by asking whether respondents had been vaccinated against COVID-19, and if yes, how many times. Depending on this answer, we then asked respondents their reasons for or against vaccination. All respondents were asked if and where they had sought information on the vaccine. We asked unvaccinated respondents about any potential incentives which would encourage them to get vaccinated, and whether they would get vaccinated if this became mandatory as the Austrian government had announced that the COVID-19 vaccination would become compulsory from February 2022. All respondents were asked if they were in favor of vaccination being made mandatory. On a five-point Likert scale, we asked respondents how worried they were about the upcoming vaccination mandate, and then asked if they would like to speak to someone about their fears and anxieties, and, if yes, with whom (e.g., an anonymous telephone hotline or a counselor).

The last section of the survey asked respondents whether they or someone they knew had contracted COVID-19, and, if yes, how severe the progression of the disease had been. Respondents were also asked what they thought of their employer's efforts to encourage vaccination.

Finally, we asked respondents whether they wanted to add further thoughts and/or comments.

Questions asking for reasons for or against getting vaccinated, sources of information, potential incentives, and the persons with whom respondents would like to speak allowed for multiple-choice answers, and also included an open-ended text field for respondents to provide additional answers. The pre-specified reasons were selected based on common answers given to healthcare workers in our research team in their daily practice, and a pre-test of the questionnaire with team members, so as to include the most commonly anticipated answers.

We included “because it is fearmongering” and “COVID-19 is not a serious disease” as two possible reasons against the vaccine. Although they seem somewhat overlapping, we nevertheless distinguished between these two items as we felt that not viewing COVID-19 as a serious disease reflects a more personal motivation, while rejecting the vaccine because it is “fearmongering” can also express a political motivation, to demonstrate resistance against the information on COVID-19 and the way it had been dispersed. The pre-test of the survey confirmed this distinction, so we opted to include both reasons despite the slight overlap.

To account for reasons not anticipated by team members and in the pre-test, we also included the option of open-ended answers.

We chose not to include a previous infection with COVID-19 as a reason against the vaccination as per the Austrian vaccination commission, a previous infection only serves as immunization for 6 months. As the vaccination had already been available for 1 year by the time we conducted this study, even previously infected respondents may have spent at least 6 months without a valid immunization status. Based on these considerations, we decided to exclude a previous infection as a reason against getting the vaccine. Respondents were however able to cite a previous infection as a reason against getting the vaccine in the open text field.

### Data analysis

Data analysis was conducted using IBM^®^ SPSS^®^ Statistics, version 26.0.0. We conducted descriptive analyses, first calculating frequencies and percentages, and, where applicable, mean, median and standard deviation. Numeric variable age was assigned into categories for inclusion in cross tabulations; for scaled questions, we also calculated top 2/bottom 2 values. We used Pearson's Chi-squared test or Fisher's exact test to determine group differences between categorical variables, and Student's t-test for continuous variables. A two-sided probability value of < 0.05 was considered significant. We used vaccination status as a dependent variable and compared it to all demographic variables (age, gender, level of education, and country of birth). Open-ended text field responses were categorized and included in the statistical analysis as additional variables.

Respondents' final thoughts and comments were analyzed qualitatively. Three independent reviewers identified common themes through iterative engagement with the responses. Findings were compared. In the case of discrepancies, discussions with two additional researchers were held to reach consensus.

## Results

In total, 625 respondents completed the survey, 36 (5.8%) *via* telephone and 589 (94.2%) *via* online survey. 73.2% (*n* = 444) of the sample identified as female, 26.4% (*n* = 160) as male and 0.5% (*n* = 3) as diverse. The age of respondents ranged from 19 to 65 years (M = 42.7, SD = 10.56). Further socio-demographic characteristics can be found in Table 1.

**Table 1:** Frequencies and percentages for demographic data.

**Demographic characteristics**	**Total** **(*n* = 625)**	**Vaccinated respondents** **(*n* = 532)**	**Unvaccinated respondents** **(*n* = 85)**
	***n* (%)**	***n* (%)**	***n* (%)**
Gender			
Male	160 (26.36)	140 (26.77)	20 (24.39)
Female	444 (73.15)	382 (73.04)	60 (73.17)
Diverse	3 (0.49)	1 (0.19)	2 (2.44)
Vaccine status			
Vaccinated once	18 (2.92)	18 (3.38)	0 (0.00)
Vaccinated twice	88 (14.26)	88 (16.54)	0 (0.00)
Vaccinated three or more times	426 (69.04)	426 (79.70)	0 (0.00)
Not vaccinated	85 (13.78)	0 (0.00)	85 (100.00)
Country of birth			
Austria	(76.02)	410 (77.80)	234 (64.63)
Not Austria	(23.98)	117 (22.20)	36 (35.37)
Level of education			
Compulsory school or lower	25 (4.30)	19 (3.82)	3 (3.85)
Apprenticeship	22 (16.67)	77 (15.49)	19 (24.36)
Leavin certification	97 (22.16)	107 (21.53)	19 (24.36)
University/ University of applied sciences	129 (56.87)	291 (58.55)	37 (47.44)

Of the 617 respondents who disclosed their vaccination status, 86.2% (*n* = 532) had received at least one dose of the COVID-19 vaccine; 13.8% (*n* = 85) were unvaccinated.

### Reasons for or against the vaccine

The primary reason for getting vaccinated given by vaccinated respondents was to protect themselves (79.6%) as well as others (74.1%), followed by the vaccine's societal importance (61.9%) and the ability to participate in social life again (47.8%). Less often cited reasons were a recommendation by employers (19.1%), physicians (7.3%), or friends and family members (6.9%), while 3.8% admitted they had “just done it” without giving it much thought. 10.9% gave an additional reason in the open-ended text field, most frequently citing pressure from their employer (3.9%).

The primary reason given by unvaccinated respondents for not getting vaccinated was fear of short or long-term side effects: 58.8% expressed uncertainty about potentially negative long-term effects; 42.4% cited negative short-term side effects (headache, fatigue, or fever) suffered by friends, family members or their patients and clients; 18.8% said they were willing to get vaccinated but were currently waiting for a different vaccine to be approved, one which they deemed safer. Other reasons given for not getting vaccinated related to the severity of COVID-19: 42.4% thought the media and politicians were fearmongering, and 35.3% said they did not trust experts who spoke out in favor of the vaccine; 29.4% stated they took a variety of protective measures (wearing a mask, washing hands, regular exercise, taking vitamins, etc.); 28.2% said they thought the government was trying to control its citizens and consequently did not want to follow the vaccine recommendations; and 22.4% expressed the belief that COVID-19 was not a serious disease.

Medical reasons for not getting vaccinated were cited less frequently: a pre-existing condition (9.4%); the wish for a child (8.2%); general vaccine skepticism (5.9%); fear of needles (1.2%); or a current pregnancy (1.2%). Recommendations against vaccination issued by friends or family (5.9%) or physicians (4.7%) were also cited. 4.7% said they had not (yet) been vaccinated because they lacked information on the subject.

43.5% offered additional reasons for not getting vaccinated, most commonly reiterating their skepticism toward the vaccine's safety and efficacy, with three respondents referring to persons who had died or suffered irrevocable damage to their health following vaccination, cases of which they knew from hearsay. Six respondents cited their general disapproval of the way the government had handled the pandemic (e.g., contradictory regulations, the distribution of COVID-19 aid packages for companies in danger of bankruptcy, or politicians' general lack of trustworthiness) as a reason against vaccination (Table 2).

**Table 2:** Reasons for or against getting the COVID-19 vaccine.

**Reason**	**n**	**%**
For getting the vaccine		
Protecting oneself	424	79.55
Protecting others	395	74.11
Societal importance	330	61.91
To participate in social life again	255	47.84
Recommended by employer	102	19.14
Recommended by physician	39	7.32
Recommended by friends/family	37	6.94
Just did it	20	3.75
Other	58	10.88
Against getting the vaccine		
Fear of long-term effects	50	58.82
Because it is fearmongering	36	42.35
Fear of short-term effects	36	42.35
No trust in experts	30	35.29
Alternative protection	25	29.41
Because the government uses it to control its citizens	24	28.24
COVID-19 is not a serious disease	19	22.35
Waiting for another vaccine	16	18.82
Pre-existing condition	8	9.41
Want to have a baby	7	8.24
Friends/family advised against	5	5.88
General rejection of vaccines	5	5.88
Lack of information	4	4.71
Physician advised against	4	4.71
Afraid of needles	1	1.18
Pregnancy	1	1.18
Other	37	43.53

Data is presented as frequency and percentage of a total n of 533 (for the vaccine) and 85 (against the vaccine).

### Sources of information

Analysis of the differences between vaccinated and unvaccinated respondents with respect to sources of information revealed significant differences for five sources: respondents who were vaccinated were more likely to read a daily newspaper (41.4 vs. 25.9%, *p* = 0.007) while respondents who were unvaccinated more often consulted the internet (37.7 vs. 25.9%, *p* = 0.025), their primary physician (27.1 vs. 16.4%, *p* = 0.017), other physicians (48.2 vs. 23.1%, *p* < 0.001), or other sources such as TV news or programs (27.1 vs. 10.3%, *p* < 0.001). Differences between the groups with respect to sources of information is shown in Table 3.

**Table 3:** Sources of information.

**Item**	**Vaccinated respondents** **(*n* = 532)**	**Unvaccinated respondents** **(*n* = 85)**	***p-value***
	***n* (%)**	***n* (%)**	
Academic articles	305 (57.33)	45 (52.94)	0.448
Employer	216 (40.60)	33 (38.82)	0.756
Daily newspaper	220 (41.35)	22 (25.88)	0.007
Internet	138 (25.94)	32 (37.65)	0.025
Non-primary physician	123 (23.12)	41 (48.24)	<0.001
Friends/acquaintances	123 (23.12)	28 (32.94)	0.051
Family	93 (17.48)	20 (23.53)	0.181
Primary physician	87 (16.35)	23 (27.06)	0.017
Colleagues	67 (12.59)	24 (28.24)	<0.001
Somewhere else	55 (10.34)	23 (27.06)	<0.001
Did not seek out information	14 (2.63)	2 (2.35)	0.881
Telephone hotline	7 (1.32)	2 (2.35)	0.459

### Mandatory vaccinations

Unvaccinated respondents were asked which incentives could make them change their mind about getting the vaccination. 72.8% said no incentive could make them change their mind, while 17.4% specified abstract concepts or systemic change, such as “if politicians lied less” or “if vaccinations were safer.” Monetary incentives were not seen as a motivator: no respondents were willing to get vaccinated for EUR 50, and only one was willing to do it for EUR 500. When asked if they would get vaccinated once it became mandatory, most respondents (74.3%) stated they would rather lose their job than get vaccinated, while 18.9% said they would only get vaccinated if this was required to keep their job (Table 4). Correspondingly, only one unvaccinated respondent (1.2%) was in favor of mandatory vaccinations compared to 64.6% of vaccinated respondents. Unvaccinated respondents were significantly more worried about the general future than vaccinated respondents (78.8 vs. 26.3%, *p* < 0.001). They were also significantly more likely to view their employers' vaccine recommendations as “manipulative” (50.6 vs. 12.4%, *p* < 0.001), while vaccinated respondents were significantly more likely to view these efforts as “supportive” (68.0 vs. 25.9%, *p* < 0.001) (Table 5).

**Table 4:** Possible vaccination incentives and response to vaccine mandate.

**Item**	**Unvaccinated respondents** **(*n* = 85)**
	***n* (%)**
Incentive	
No incentive could change mind	67 (72.83)
Abstract concepts/systemic change	16 (17.39)
Monetary incentive (EUR 50)	0 (0.00)
Monetary incentive (EUR 50)	1 (1.09)
Response to vaccine mandate	
Would rather lose my job than get vaccinated	55 (74.32)
Would only get vaccinated if the alternative was losing my job	14 (18.92)
Would get vaccinated if it was mandatory	5 (6.76)

**Table 5:** Feelings related to vaccine mandate and employer information.

**Item**	**Vaccinated respondents** **(*n* = 528)**	**Unvaccinated respondents** **(*n* = 84)**	***p-value***
	***n* (%)**	***n* (%)**	
Opinions on vaccine mandate			
In favor of vaccine mandate	296 (64.63)	1 (1.23)	
Worried about the general future	140 (26.32)	67 (78.82)	<0.001
Perception of employer information			
Manipulative	66 (12.41)	43 (50.59)	<0.001
Supportive	362 (68.05)	22 (25.88)	<0.001

### COVID-19 infections

Respondents who were unvaccinated at the time the data was collected had already been infected with COVID-19 more often than vaccinated respondents (28.6 vs. 12.7%, *p* < 0.001), although they were only slightly more likely to have been hospitalized (one case in each group).

No significant differences could be gleaned from the comparative analysis of respondents who were vaccinated and those who were unvaccinated with respect to whether they knew someone who had been infected with COVID-19, and if so, how severe the course of the disease had been for that person (Table 6).

**Table 6:** COVID-19 infection.

**Item**	**Vaccinated respondents** **(*n* = 528)**	**Unvaccinated respondents** **(*n* = 84)**	***p-value***
	***n* (%)**	***n* (%)**	
Self			
Had COVID-19, home quarantine	6 (12.50)	23 (27.38)	
Had COVID-19, hospitalized	1 (0.19)	1 (1.19)	
Had COVID-19, ICU	0 (0.00)	0 (0.00)	
Did not have COVID-19	461 (87.31)	60 (71.43)	<0.001
Someone they know			
Had COVID-19, home quarantine	157 (29.85)	21 (25.00)	0.364
Had COVID-19, hospitalized	99 (18.82)	19 (22.62)	0.415
Had COVID-19, ICU	135 (25.67)	17 (20.24)	0.285
Know no one who had COVID-19	135 (25.67)	27 (32.14)	0.214

## Discussion

This extensive study involving over 600 participants—all employees of a healthcare NGO in Austria—surveyed employee motivation behind the acceptance or refusal of a COVID-19 vaccine, the information they had gathered in order to make this decision, and in particular the role of employer incentives and measures in their decision-making process. Furthermore, the study assessed the impact of country-wide policy discussions such as potential vaccine mandates on individual vaccination decisions. Below, we discuss the implications of our findings for COVID-19 vaccination motivation in general, and with a focus on the role of employers as mediators in employee decision-making processes when it comes to public health goals such as vaccination.

Regarding the decision to become vaccinated, our study found no major differences between our study population of a healthcare NGO's employees compared to the rest of the Austrian population (19). The overriding factor behind the decision to get vaccinated was personal protection and to protect others, and to similar degrees. The study had hypothesized that protecting others might score higher amongst nursing and social care employees than in the rest of the population as many of the study participants were either healthcare workers themselves and had direct patient contact, or, as a result of working within the organization, had seen firsthand how COVID-19 had endangered some vulnerable people in the NGO's care. However, this assumption was not confirmed by the data. This suggests the study might add to the growing body of literature indicating that patient care and care work more generally are problematic predictors for vaccine decision-making (20–22). It should be noted that not all study participants had direct contact with vulnerable patients, and, for reasons of anonymity, additional data was not acquired to establish such differences. Therefore, any correlation between getting vaccinated against COVID-19 to protect others and working in direct contact with patients cannot be statistically confirmed. In open answers given by participants, however, references to work with clients/patients was repeatedly specified as a (co-)motivation for vaccination. In some cases, this was even given as the primary motivation where the study participants did not feel they themselves needed protection from an infection in the form of a vaccine. More in-depth research, especially on the quality of such motivation, is needed.

19.1% of participants said that their employer's recommendation had at least played a partial role in getting vaccinated. In the open answers, an additional 3.9% said they had felt pressured by their employer to get vaccinated, highlighting how employers can and do have a measurable impact on public health. This reflects recent findings, such as a large-scale survey by Fishman et al. which showed that employer mandates had a significant effect on vaccination decision-making (23). Other authors have also examined the impact of other non-“crime and punishment” measures, such as incentives and the reduction of what Njoku et al. (24) call “structural barriers,” including paid leave (25), easy access to non-traditional vaccination locations (26), or administrative facilitations by employers (27), and identified their incentivizing potential. Our study concurs with these results, confirming that employers can play an important role as mediators in vaccine decision-making and can, indeed, be regarded as a resource in public health policies for future vaccine rollouts. However, more detailed research on the quality of such actions is necessary, especially in terms of “softer” incentives and mediating motivators, such as (mis)trust in the employer (11, 12).

This is particularly pertinent considering that, at the time the study was conducted, there were heated political and public debates on whether to instate a vaccine mandate for COVID-19 for the general population, or at least for people in the healthcare sector, with both policies directly affecting most of our study participants. Our study found that this would have little effect on peoples' reported behavior, in some cases even making the participants more determined not to get vaccinated as a means of expressing their own free will. While the study merely asked what people would do if a vaccine mandate were to be instituted (rather than verifying that these claims were being put into practice), this outcome is still valuable for assessing the possible effects of a vaccine mandate, whether a universal mandate or only for those working in the healthcare sector. In open questions, the participants' reasons for refusing to get vaccinated, even with a mandate in force, became apparent: many voiced grave concerns regarding their own health, even stating that they believed their life to be in acute or long-term danger if they were to receive the vaccine. Consequently, while not a desirable outcome, losing their job or having to pay a fine for defying a vaccine mandate seemed a comparatively small price to pay to protect one's health or even life. As one participant said, “What good will a job do me if I'm dead?”.

Furthermore, some respondents saw not getting vaccinated as an act of civil disobedience, giving them a means of expressing their resistance to a political system that, according to them, encroached on their physical and health autonomy. While pressure acted as a motivator behind some respondents' decision to get vaccinated, high-stakes “crime and punishment” measures may have a significant, detrimental effect on precisely those at whom they are targeted. “Softer” measures, such as providing opportunities to receive more information or easy access to on-site vaccination would seem preferable. In many cases external factors such as distrust in and resistance to (governmental) power will trump any measures taken by the employer.

Similarly, the main reason given by study participants (as in the population as a whole) for refusing a COVID-19 vaccination is mistrust in the vaccine itself, combined with disbelief in the information provided by medical professionals, the government, or both. The fact that many of our participants have medical training or above-average knowledge of medical issues as a result of their profession did not alter this outcome. Instead, people used their expert knowledge to justify their personal choice not to be vaccinated, using arguments and methods that can be ascribed, at a general level, to scientific practice: such as in-depth research, consulting medical journals, citation, evaluating the quality of sources according to factors perceived to determine “scientificity,” or postponing their decision based on risk assessments, weighing the risk of a COVID-19 infection against the health risk they associate with the COVID-19 vaccine [see also (28)]. The study found fewer differences than expected between vaccinated and unvaccinated respondents in terms of the sources of information participants reported to have accessed to inform themselves about the COVID-19 vaccination. Both those who opted for the vaccine and those who did not reported having referred to daily media outlets, the internet and (primary) physicians when making their decision. In the open answers, several participants who opted against the vaccine stressed that they had used “scientifically solid sources of information,” such as physicians' advice and scientific journal articles to make their choice. Other studies about attitudes toward vaccines amongst medical professionals in Austria have recently produced similar findings, noting that information alone does little to persuade people who are hesitant to vaccinate, especially healthcare experts, if more structural factors are not taken into account (16). Moreover, people may decide based on their existing world or political views, or on a primarily emotional basis, that they will or will not get vaccinated, and then both seek out and interpret medical information accordingly to substantiate their decision. Consequently, increased health literacy certainly can lead to increased patient empowerment (29), as individuals feel they have all necessary information available to make health decisions best suited to their needs, and, in fact, might objectively increase the level of understanding regarding the consequences of health decisions (30). However, while low health literacy is associated with poorer health outcomes (31), the opposite is not necessarily true, as our study, and others, have found. Thus health literacy does not necessarily guarantee a different behavioral outcome (32).

Despite widespread similarities in the sources of information, the differences shown in the data are indeed meaningful. Respondents who were not vaccinated reported using internet sources more frequently, in addition to or instead of daily media outlets such as newspapers and TV. This could indicate that this group decided to gather their own information rather than merely consuming mainstream media sources. This accords with the questioning of public authorities such as politicians and researchers, leading them to seek out other sources of information. This does not necessarily mean that unvaccinated study participants consumed questionable, lower-quality sources of information than their vaccinated counterparts. Instead, it could also indicate that they consumed similar sources, such as scientific journal articles, as noted above, but drew different conclusions from what they read. In an open answer, for example, one participant gave the correct efficacy rate for the COVID-19 vaccine in question but concluded that this did not provide sufficient protection from infection to outweigh the possible risks of vaccination. Overall, most study participants—both vaccinated and unvaccinated—appeared to be well informed and to have made a conscious decision.

Another finding in this context is the role of physicians and other health professionals in shaping opinions. Both vaccinated and unvaccinated study participants claimed to have followed their physician's advice (not) to receive a COVID-19 vaccine, with general practitioners having been named more frequently in this context by non-vaccinated individuals. This showcases the crucial role of health professionals as opinion leaders and mediators in the decision-making process. From the perspective of an employer wanting to motivate employees to partake in public health measures such as vaccinations, this indicates that building trust between occupational health physicians and employees could significantly impact the success of health measures.

## Conclusion

It should be reiterated that many of our respondents work as caregivers and thus have an above-average level of medical expertise. As a result of their employer's extensive dissemination of information on the COVID-19 vaccine, they had also had manifold opportunities to educate themselves on the benefits of the vaccine. This, together with the fact that respondents work with and care for vulnerable and at-risk groups, led to a feeling of shame in many of those respondents who had decided not to receive the vaccination (yet). The phone interviews in particular made this palpable: many as-yet unvaccinated respondents expressed relief that this study finally gave them the chance to voice reasons for their hesitancy in a judgment-free environment. This phenomenon has been dubbed “unspoken vaccine hesitancy” by Heyerdahl et al. (33), who state that “especially among healthcare workers, merely voicing vaccine-related concerns entails a risk of being lectured, mocked, stigmatized, or labeled as conspiracy theorists and ‘anti-vaxxers”’ (p. 1). In this sense, this study also provided a space for people to voice such opinions, highlighting that vaccine hesitancy or vaccine refusal are complex phenomena and take place on a spectrum more complicated than a mere yes/no choice (34, 35). In the same sense, people who choose not to vaccinate are not “beyond help,” and incentivizing measures can indeed make a difference—but there is no “one size fits all”-solution.

Providing holiday coupons, financial incentives, or entering unvaccinated people into a lottery to win attractive prizes—measures which were discussed or implemented in Austria (36)—are not panaceas, nor often a sufficiently weighty counterbalance for those truly concerned about endangering their wellbeing with a vaccine. Employers have the means to mediate public health decision-making by providing employees with information, but vaccination decision-making is a more complex process that involves public debates, world views, political influences, and the uptake of the information provided. As a result of the NGO's efforts to educate and incentivize, the vaccination rate among our respondents was 86.2% and thus already higher than that of the Austrian general population which was around 75% at the time (6). Nevertheless, a crucial minority of NGO employees had decided against getting vaccinated. The NGO's efforts were an important contributor to vaccination decision-making, but not sufficient to achieve a full vaccination rate among its staff. We conclude that employers need to be taken seriously as mediators for public health decision-making, moving policy measures beyond an individualized view of health choices and health literacy toward more structural, systemic, and community-based efforts (37). Employer incentives should be thought of as a “connected effort,” one that intersects with a network of reasons for vaccine decision-making. This present study has pointed to informational incentives, measures tailored to the workforce (e.g., healthcare institutions need to take the professional client relationship into account), the potential role of occupational medicine as “in-house” opinion leaders—assuming trusting relationships can be established—and the importance of a varied approach which goes beyond “crime and punishment” mandates.

Overall, there cannot be a one-size-fits-all approach to complex health decision-making, especially in times of crisis. If a common goal is to be achieved, measures to achieve this goal need at least to try to bring everyone into the fold.

## Limitations

This study is not without limitations. Firstly, cross-sectional studies have some general disadvantages: they depict only one moment in time, and it is difficult to make causal inferences (38). Secondly, we must note the unique profile of our study participants: as our participants work for a nursing and social care NGO, we argue that the profession in which they work already shapes their perception of matters related to public health, such as a global pandemic. We therefore cannot use our data to draw conclusions for the Austrian population as a whole. Even among nursing and social care employees, our respondents represent a minority: before the beginning of our study, their employers had already disseminated information about the vaccine, recommended the vaccine, and even implemented individual mandatory consultations with a physician for all unvaccinated employees. Other nursing service providers whose respondents were not included in our study had not taken similar measures to inform and encourage their employees to get vaccinated.

## Data availability statement

The raw data supporting the conclusions of this article will be made available by the authors, without undue reservation.

## Ethics statement

The studies involving human participants were reviewed and approved by Ethical Review Board for the Viennese Hospitals in the Vinzenz Holding. The patients/participants provided their written informed consent to participate in this study.

## Author contributions

TW-T, MK-P, SV-K, and EK conceived of the study. SK, ME, AT, MG, and A-KR conducted the telephone interviews. A-KR programmed the survey online and performed the statistical analysis. ME and A-KR drafted the final manuscript and performed all necessary revisions. All authors participated in the design of the study. All have authors read and approved the final manuscript.

## Conflict of interest

The authors declare that the research was conducted in the absence of any commercial or financial relationships that could be construed as a potential conflict of interest.

## Publisher's note

All claims expressed in this article are solely those of the authors and do not necessarily represent those of their affiliated organizations, or those of the publisher, the editors and the reviewers. Any product that may be evaluated in this article, or claim that may be made by its manufacturer, is not guaranteed or endorsed by the publisher.

## Footnotes

^1^However, after it was discovered that the Omicron variant proved less of a threat than Delta, the vaccine mandate was never enforced and was quietly discarded in June 2022 (18).

^2^ https://www.sozialministerium.at/Themen/Gesundheit/Medizin-und-Gesundheitsberufe.html
